# Prevalence and Associated Factors of Psychological Distress among Nurses in Public Hospitals, Southwest, Ethiopia: A cross-sectional Study

**DOI:** 10.4314/ejhs.v31i6.21

**Published:** 2021-11

**Authors:** Alemayehu Sayih Belay, Melak Menberu Guangul, Wondwossen Niguse Asmare, Gebremeskel Mesafint

**Affiliations:** 1 Department of Nursing, College of health sciences, Mizan-Tepi University, Mizan-Teferi, Ethiopia; 2 Department of Psychiatry, College of medicine and health sciences, Bahir Dar University, Bahir Dar, Ethiopia

**Keywords:** Factors, Nurses, Prevalence, Psychological distress, Ethiopia

## Abstract

**Background:**

Psychological distress is a state of emotional suffering and also characterized by somatic symptoms. Health care workers more prone psychological distress than general population. However, little attention was paid on psychological distress among nurses particularly in Ethiopia. Therefore, this study aimed at assessing the prevalence of psychological distress and its' associated factors among nurses in public hospitals, Southwest Ethiopia.

**Method:**

An institutional-based cross-sectional study was conducted from February 1^st^, 2018 to April 1^st^, 2018. All 282 eligible nurses in the selected public hospitals were enrolled. Data was collected using the predesigned tool like Self-Reporting Questionnaire version 20. Data were entered using EPI INFO version 7 and was exported to statistical packages for social science (SPSS) version 21.0 for analysis. Logistic regression analysis was employed and variables with a P-value of < 0.05 were considered as statistically significant.

**Result:**

A total of 282 eligible nurses were enrolled in the study with mean age of 28.71 [SD ±7.047]. The prevalence of psychological distress among nurses was 78(27.7%). Predictor variables like; nurses with job title of staff nurse, less working experience, poor interaction with staffs, fatigue, poor social support, perfectionism, and insomnia were more prone to develop the psychological distress.

**Conclusion:**

The study revealed that a considerable proportion of nurses had psychological distress. Therefore, it needs to develop psychological support strategies to improve the mental health resilience of nurses.

## Introduction

Psychological distress (PD) is broadly defined as a state of emotional suffering characterized by symptoms of depression (e.g., loss of interest; unhappiness; desperateness) and anxiety (e.g., restlessness; feeling tense). It is also characterized by other somatic symptoms like; insomnia, headaches, and lack of energy that are likely to vary across different areas (1).

According to the Diagnostic and Statistical Manual of Mental Disorders, Fifth Edition (DSM-5), psychological distress defines as “undifferentiated group of symptoms ranging from anxiety and depression symptoms to functional impairment, personality traits (confusing, troubling), and behavioral problems (2).

Psychological distress is also considered as a transient (not long-lasting) phenomenon that is associated with specific stressors and characterized by disturbances in sleep, fluctuations with the eating pattern, headache, constipation, diarrhea, chronic pain, provoked to anger frequently, excessive tiredness, forgetfulness and memory problems, and no longer finding pleasure in sex. It typically diminishes/ vanishes when either the individual adapts to the stressor, or the stressor is removed (3).

Psychological distress among health care workers is common, especially among nurses (4). Because they were providing continual care for those chronically ill patients, where the nature of the job by itself facing nurses to an increasing workload and risk of infection to different communicable diseases (5). Therefore, they are susceptible to psychological distress and other complex emotional reactions. Furthermore, the mental health problems of nurses would impair their cognitive functioning, attention to health procedures, and clinical decision-making capacity. In general, their psychological distress will have an impact on the quality of health services and patient satisfaction (6). High expectations, lack of time and skills, and poor social support can also cause occupational stress and psychological distress which can lead to post-traumatic stress disorder. This is because nurses with no adequate skills towards different nursing procedures, poor interaction and support among different staffs, poor sleep pattern, and high expectation with poor achievement leading to dissatisfaction among nurses which finally causes psychological distress (7).

Psychological distress also increases the risk of heart disease and decrease immunity, thus increases the risk to different illness (8). It also affects the quality of life and work, including overall well-being, social relationships, and family life. Moreover, this can result in higher turnover, work absences, early withdrawal, and lower quality of health services (8, 9).

The prevalence of psychological distress is ranged between 5% and 27% in the general population (10–14), while the study conducted in Nigeria revealed that it raises up to 44.1% among nurses (15). Therefore, the high prevalence of psychological distress among nurses due to poor interaction among staff and workload also contributes to high turnover and decrement of job satisfaction which become a global issue (16).

In Ethiopia, the lack of reliable data on psychological distress (PD) often hampers officials from planning for preventive actions. In Ethiopia, the burden of psychological distress (PD) remains challenging among health care workers. However, the Ethiopian Ministry of Health (MOH) collaborates with different programs like Mental Health Gap Action Program (mhGAP), and Program for Improving Mental Health Care (PRIME) to scale-up the mental health services (17).

Since nurses contribute a major role in the provision of direct care for the patients in every health institution twenty-four hours a day, and seven days a week (18), assessing for the prevalence of psychological distress and its associated factors in public hospitals of southwest Ethiopia will be helpful for policymakers to provide the timely intervention.

Even though few studies on the prevalence of psychological distress in Ethiopia have been previously done, those studies are conducted among students (19), and family caregivers (20). Therefore, this study aimed to assess the prevalence and associated factors of psychological distress among nurses in public hospitals, southwest Ethiopia.

## Materials and Methods

**Study design and period**: An institutional-based cross-sectional study design was carried out at public Hospitals from February 1^st^, 2018 to April 1^st^, 2018.

Study setting

The study was conducted in public hospitals located in zones of Southwest Ethiopia. These are Mizan-Tepi University Teaching Hospital, Gebretsadik Shawo General Hospital, and Tepi General Hospitals, which are located in Bench-Maji Zone, Kefa Zone, and Sheka Zone respectively. Mizan-Tepi University Teaching Hospital has located 561 km from the capital city Addis Ababa and 844 km from the regional city Hawassa. Gebretsadik Shawo Hospital is found in Bonga town and located 464 km from Addis Ababa and 739 km from the regional city Hawassa. Tepi General Hospital is found in Tepi and located 611 km from Addis Ababa. All of these hospitals have immunization department, delivery ward, inpatients wards, outpatient departments, antenatal and post-natal clinic.

Currently, Mizan-Tepi University Teaching Hospital is expected to provide service for more than 829,000 populations. While Gebretsadik Shawo General Hospital and Tepi General Hospitals are expected to provide care for more than 500,000 populations. Mizan-Tepi University Teaching Hospital, Tepi General Hospital, and Gebretsadik Shawo General Hospital have 106, 94, and 82 nurses respectively whose work experience is one or more years.

**Study participants**: To be included, participants had to fulfill the following criteria: (1) have a minimum of a year of work experience in the same workplace or a similar health setting, (2) age 18 years and older, and (3) being nurse and working in public hospitals. But those who were critically ill and unable to give a response during data collection time were excluded. All the 282 nurses who were available during the data collection period and who fulfill the inclusion criteria were included in the study.

**Data collection tools and procedure**: The data was collected using a self-administered questionnaire by six trained nurse professionals and supervised by three health officers. One day of training was given by principal investigators for the data collectors and supervisors on the objective of the study, method data collection, and its' supervision. The questionnaire was pretested among 5% of the sample size in health centers which were out of the study settings. The coherence and skipping pattern of the questionnaire were corrected after the pretest. Then, the actual data collection was conducted by distributing the self-administered questionnaire for the eligible participants to be filled after the objective of the study was properly explained. Finally, the questioner was checked for its completeness on daily basis.

For psychological distress (anxiety, depression, and somatization), the Self-Reporting Questionnaire version 20 (SRQ 20) was used, which was developed by World Health Organization (WHO) for low and middle-income countries including Ethiopia. It has a “YES” or “No” questions and can be used as a self-administered or interviewer-administered questionnaire. SRQ 20 is a 20 item questionnaire commonly used to screen anxiety, depression, and somatization symptoms. We used the cut-off point 8 based on the finding from the validation study of SRQ-20 which gave the highest sensitivity and specificity (21).

To assess social support, Oslo Social Support Scale was used. Oslo Social Support Scale Score is ranged from 3–14 with a score of 3–8 = poor social support; 9–11 = intermediate social support; and 12–14 = strong social support (22).

Sleep disturbance was measured by the Athens insomnia scale (AIS). The AIS is an 8-item self-reported questionnaire that indicates insomnia within the past month and scores each ranging from 0 to 3 (0 score equals better and 3 is worst). The total score greater than or equals to 6 indicates insomnia (23).

**Data analysis**: The data was entered using EPI INFO version 7 and was exported to statistical packages for social science (SPSS) version 21.0 for data cleaning and analysis. Bivariate logistic regression was done to see the degree of association between independent variables (socio-demographic, organizational related factors, psycho-social related factors), and dependent variable (psychological distress) were assessed. Variables with a P-value of <0.05 were recruited for multivariate analysis to control the effects of confounding. Finally, the results considering a confidence level of 95% and a Pvalue of <0.05 were taken as strong predictors for psychological distress. The results were presented in the form of tables and summary statistics.

**Ethics approval**: The study was approved by the research standing committee of Mizan Tepi University, college of health science with a reference number CHS/0246/15/19. In addition to this, a permission letter was taken from each hospital administration office. After explaining the objectives of the study, written consent was obtained from each study participant. Then after data was collected with strict privacy and assuring confidentiality.

## Results

**Socio-demographic characteristics of the respondents**: A total of 282 eligible nurses were included in the study with a 100% response rate. Among the respondents, most of 125 (44.3%) were in the age range of 25–29 years with a mean age of 28.71 [SD ±7.047], more than half of participants 144 (51.1%) were female, 91 (32.3%) were Amhara by ethnicity, 120 (42.6%) were currently single, 158 (59.6%) had diploma educational status, 157 (58.6%) were orthodox religion members followed by protestant 34.3%. Concerning the respondents' working years of experience in the current health institution, most 138 (48.9%) of them had 1–3 years of experience (Table 1).

**Table 1:** Socio-demographic characteristics of participants in public hospitals, South West Ethiopia, 2017/18

Variable	Frequency	Percent
Sex		
Male	138	48.9
Female	144	51.1
Age in year		
20–24	73	25.9
25–29	125	44.3
30–34	35	12.4
35–39	25	8.9
>=40	24	8.5
Marital status		
Single	120	42.6
Married	106	37.6
Divorced	23	8.2
Widowed	17	6.0
Separate	16	5.7
Educational status		
Diploma nurse	168	59.6
Bsc nurse	114	40.4
Religion		
Orthodox	165	58.5
Protestant	89	31.6
Muslim	19	6.7
Catholic	9	3.2
Ethnicity		
Amhara	91	32.3
Kaffa	53	18.8
Oromo	42	14.9
Sheka	33	11.7
Bench	22	7.8
Tigre	17	6.0
Dawaro	9	3.2
Others *	15	5.3
Job title		
Staff Nurse	232	82.3
Specialist Nurse	23	8.2
Head Nurse	27	9.6
Year Of Experience		
<3	138	48.9
3–5	66	23.4
6–10	56	19.9
11–15	13	4.6
>15	9	3.2

* Gurage, Sidama, Wolayeta

**Organizational and psycho-social related variables**: Among all respondents, more than half of respondents did not get sufficient reward for their work 160 (56.7%), the majority 221 (78.4%) of nurses had good interaction with their co-workers, 237 (88.4%) of them were practiced caring of dying patients, and more than half 150 (53.2%) of the participants were planning to leave their working environment in the near future.

More than half of respondents 155 (55.0%) had intermediate social support, whereas 65 (23%) and 62 (22%) of them had low and strong social support respectively. Of the total participants, about 107 (37.9%) had fatigue syndrome, 244 (86.5%) had a problem with perfectionism, and 51(18.1%) had a major problem with sleep (Table 2).

**Table 2:** Organizational and psycho-social related variables of participants in selected public hospitals, South West Ethiopia, 2017/18

Variable	Frequency	Percent
Having insufficient reward		
Yes	160	56.7
No	122	43.3
Having good interaction		
with other staffs	221	78.4
Yes	61	21.6
No		
Caring of dying patients		
Yes	248	87.9
No	34	12.1
Intending to leave working		
environment	150	53.2
Yes	132	46.8
No		
Fatigue		
No	175	62.1
Yes	107	37.9
Social support		
Poor	65	23.0
Intermediate	155	55.0
Strong	62	22.0
Problem with perfectionism		
No	38	13.5
Yes	244	86.5
Sleeping problem		
Normal sleep	131	46.5
Minor problems with sleep	100	35.5
Major problems with sleep	51	18.1

**Prevalence of psychological distress**: The prevalence of psychological distress among nurses was 78(27.7%) with 95% CI (22%, 33%).

**Factors associated with psychological distress**: Based on bivariate analysis variables like; age, job title, year of experience, interaction with staff, fatigue, social support, perfectionism, and insomnia were the factors found to be significantly associated with psychological distress.

After adjusting for possible confounders with multivariate analysis; job title, work experience, interaction with staff, fatigue, social support, perfectionism, and insomnia were found to be strong predictor variables for psychological distress.

The odds of having psychological distress were almost 10 times more likely in those participants with the job title of a staff nurse as compared with a head nurse [AOR= 10.13, 95% CI (1.85, 55.41)]. Participants whose working experience were 6–10 years were almost 5 times more likely to have psychological distress than those participants with experience of 1–3 years [AOR= 4.98, 95% CI (1.59, 15.568)]. Nurses with good interaction with other staff members were 30.5% times less likely to be psychologically distressed as compared with their counterparts [AOR= .305, 95% CI (.117, .796)].

Participants with fatigue were 2.5 times more likely to have psychological distress as compared with their counterparts [AOR= 2.54, 95% CI (1.072, 6.02)]. Those participants with intermediate social support were 18.9% times less likely to have psychological distress than those with poor social support [AOR= .189, 95% CI (.075, .474)].

Besides, participants with perfectionism were almost six times more likely to develop psychological distress than their counterparts [AOR= 5.697, 95% CI (1.449, 22.39)]. The odds of having psychological distress were almost 4 times more likely in those participants with a minor problem with sleep as compared with those participants with normal sleep [AOR= 3.82, 95% CI (1.52, 9.579)] (Table 3).

**Table 3:** Bivariate and multivariate analysis of predictor variables for psychological distress in selected public hospitals, South West Ethiopia, 2017/18

Variables	Psychological Distress		COR (95%CI)	AOR (95% CI)

	No N (%)	Yes N (%)		
Age				
20–24	53(72.6)	20(27.4)	1.00+	1.00+
25–29	96(76.8)	29(23.2)	.801(.413–1.551)	.410(.153–1.096)
30–34	30(85.7)	5(14.3)	.442(.150–1.297)	.048(.008–1.313)
35–39	9(36.0)	16(64.0)	4.71(1.795–12.37)*	4.640(.891–24.160)
>=40	16(66.7)	8(33.3)	1.325(.491–3.574)	.072(.005–1.048)
Job title				
Staff Nurse	163(70.3)	69(29.7)	5.291(1.220–22.957)*	10.125(1.85–55.41)*
Specialist Nurse	16(69.6)	7(30.4)	5.469(1.007–29.701)*	2.917(.311–27.352)
Head Nurse	25(92.6)	2(7.4)	1.00+	1.00+
Year of Experience				
<3	105(76.1)	33(23.9)	1.00+	1.00+
3–5	54(81.8)	12(18.2)	.707(.338–1.479)	2.148(.753–6.126)
6–10	32(57.1)	24(42.9)	2.386(1.236–4.61)*	4.980(1.59–15.568)*
11–15	5(38.5)	8(61.5)	5.091(1.56–16.63)*	6.081(.419–88.241)
>15	8(88.9)	1(11.1)	.398(.048–3.298)	4.256(.122–147.88)
Good Interaction with staffs				
Yes	172(77.8)	49(22.2)	.314(.174–.570)*	.305(.117–.796)*
No	32(52.5)	29(47.5)	1.00+	1.00+
Fatigue				
No	135(77.1)	40(22.9)	1.00+	1.00+
Yes	69(64.5)	38(35.5)	1.859(1.094–3.159)*	2.540(1.072–6.02)*
Social support				
Poor	33(50.8)	32(49.2)	1.00+	1.00+
Intermediate	123(79.4)	32(20.6)	.268(.144–.500)*	.189(.075–.474)*
Strong	48(77.4)	14(22.6)	.301(.139–.649)*	.919(.291–2.902)
Perfectionism				
No	33(86.8)	5(13.2)	1.00+	1.00+
Yes	171(70.1)	73(29.9)	2.818(1.058–7.505)*	5.697(1.449–22.39)*
Insomnia				
Normal sleep	119(90.8)	12(9.2)	1.00+	1.00+
Minor problems with sleep	67(67.0)	33(33.0)	4.884(2.365–10.088)*	3.82(1.52–9.579)*
Major problems with sleep	18(35.3)	33(64.7)	18.181(7.960–41.526)*	21.24(12.82–92.71)*

* Adjusted for all significant variables at p <0.05, COR= crude odds ratio AOR= adjusted odds ratio, CI = confidence interval

+ = Reference Category

## Discussion

This study aimed at assessing the prevalence of psychological distress and associated factors among nurses. In addition to this, the findings of this study will have a significant role in overcoming the problems associated with psychological distress among nurses, like; job dissatisfaction and lack of energy which negatively affects their perception of the quality of life and health services in the workplace and the relationship with the patient and family (24).

In the current study, the prevalence of psychological distress was 27.7%, which was lower prevalence as compare with the study conducted in Nigeria was 44.1% (15). The possible reason for this difference might be due to the difference in the study setting, study population, tools, and methodological differences. For instance, in a study conducted in Nigeria, the tool named General Health Questionnaire (GHQ-12) was used whereas, the Self-Reporting Questionnaire version 20 (SRQ 20) was used in our study.

In contrast to the other study, this study was found to be higher compared with the studies conducted in other areas, where in Norwegian 13% (25), and in Sri Lanka 21% (26). This discrepancy might be for the reason that there might be a difference in a study setting, study population, tools, and methodological differences. Another possible explanation might be due to the nature of their work that nurses are more prone to experience psychological distress than the general population in the community. It is also might be the difference of availability of standard medical care environment, sociocultural belief and life style of the nurses compared with this study.

Different independent predictor variables associated with psychological distress were identified in this study.

In this study, the job title was found to be significantly associated with psychological distress, where those staff nurses were more likely to develop psychological distress than head nurses. This finding is found to consistent with the study conducted in Australia (27). This might be due to the fact that those nurses in the position of staff nurse may experience more distress and less job satisfaction because they spend more time with patients. Conversely, nurses working in managerial positions, generally have more professional experience, a higher level of education, and a good monthly income, which can help them to cope with different stressors and also increase job satisfaction (28).

Work experience was also found to be significantly associated with psychological distress. Participants with a long duration of work experience were more likely to have psychological distress than those participants with a short duration of work experience. This finding also noted in London that year of work is associated with psychological distress (29). This might be explained by those nurses who worked for a long duration of time in the health setting are more subjected to emotional exhaustion, lack of energy, loss of interest, restlessness, feeling tense, and general fatigue which in turn leads to the experience of psychological distress (30).

In this study, good interaction of nurses with other staff members was less likely to be psychologically distressed as compared with their counterparts. This finding is in line with the study conducted in Virginia, USA (31). This is due to the fact that nurses with good interaction among staff and family members have a chance to discuss and seek solutions for the issues which interfere with their stability and motivation for work. In contrast to this, poor interaction can negatively affect their physical as well as mental health (32).

In this study, fatigue among nurses was considered as a significant variable for psychological distress. This is supported by a study from Rhine-Main-Region, western Mid-Germany (33). This might be for a reason that nurses with fatigue are commonly experienced with a devastating sense of drowsiness, lack of energy, and impaired cognitive and/or physical functioning, which may lead to a health problem, decrement of performance, increased risk of injury/accident, and low personal achievement, which possibly experience psychological distress. Additionally, fatigue among nurses can cause them to behave in ways that are out of character, poor performance at work, and inability to keep personal or work commitments which will probably experience psychological distress (34).

This study also found that poor social support was another factor associated with psychological distress. This finding is also supported by the study conducted in China (35). A plausible explanation for this could be the fact that nurses with good social support can have good mental and physical health, where this leads to the good cognitive function and performance of concentrated health care procedure which finally increases the quality of health care services. Moreover, good social support helps to reduce their problematic behaviors, thus enhancing their adjustment and reducing adverse effects on their day to day work related activities and psychological state (36).

Besides, this study investigated that participant with perfectionism was a predictor variable for psychological distress. This finding was supported by the study conducted in the United States Western University, USA (37). This is due to the fact that perfectionism is a driving force of negative stress in some nurses. Because perfectionist nurses who were more concerned about faults, obsessed with a higher quality of work, and had more worries about their capabilities regarding patient care are more prone to be psychologically distressed (38).

Insomnia was also another important predictor variable in this study. This is supported by the study conducted in Finland (39). The reasonable justification could be the fact that nurses with sleep problems become disoriented or lack of concentration for their work, which results in an increase in medical errors, and further, it increases the likelihood of subsequent psychological distress (40).

Even though this study contributes as an input for the policymakers towards the decrement of the psychological distress among nurses, it has its own limitations. First, since a cross-sectional study design was implemented, it can't establish a cause-effect relationship between the predictor variables and dependent variables. Second, the tool used to assess psychological distress was not validated in Ethiopia. Third, this study didn't focus on factors related to clinical intervention and treatment given among nurses with psychological distress. Finally, other health professionals were not included, which limited us not to compare nurses with other health professionals.

In conclusion, this study revealed that a considerable proportion of nurses had psychological distress. Job title, work experience, interaction with staff, fatigue, social support, perfectionism, and insomnia were found to be strong predictor variables for psychological distress. Therefore, concerned bodies at different health sectors should give attention towards the promotion of strong social support among nurses, upgraded working status, and good interaction with other staff members in order to mitigate the problems associated with psychological distress. Moreover, stress reduction strategies has to be implemented to prevent and improve problems related to insomnia.
